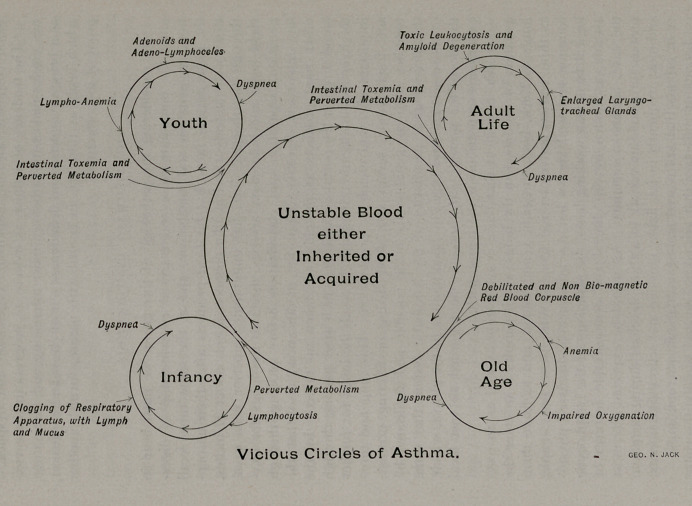# The Pathology of Asthma, with Special Reference to Its Vicious Circle1Read at the 53d annual meeting of the American Medical Association, in the section on pathology and physiology, at Saratoga, June, 1902.

**Published:** 1904-01

**Authors:** G. N. Jack

**Affiliations:** Buffalo, N. Y.


### The Pathology of Asthma, with Special Reference to Its Vicious Circle.[1]

G. N. JACK, M. D., Buffalo, N. Y.

As THE circulation of the blood through the body was slow in being discovered, so also is its complexity, function, physiology and pathology equally slow in being determined. It therefore is not strange that the pathology of one of our most anciently recognised, torturous, dreaded and surprisingly common diseases, whose pathology as can now be shown lies positively in the blood, should be reserved for the present age.

1. Read at the 53d annual meeting of the American Medical Association, in the section on pathology and physiology, at Saratoga, June, 1902.

While there is no constant positively demonstrable pathognomonic asthmatic blood lesion, yet by repeated analyses of the blood of an individual asthmatic, before, during and after an attack, together with an intelligent, comprehensive and close observation of the clinical features of the case, combined with a mortaring in of all the facts thus gained by careful and repeated analyses of the sputum, urine and stool, an impregnable and imperishable wall of facts is constructed, that will ever stand in bold declaration of the blood origin of asthma, against all the theories of the past or future, that have been, or may be constructed.

It will be seen, then, that asthma is not a disease by itself having a well established entity, but that it is only a symptom, a part of a vicious circle, or an abnormal biochemical and complex pathological process, originating usually in the intestinal canal, through a long standing intestinal indigestion and toxemia, with faulty absorption and metabolism, producing a toxic or lymphogenous chyle that generates an unstable blood, characterised by its extremely varied, numerous and alarming paroxysmal, morphologic changes often alternating between a lymphocytosis, an intestinal toxemic leukocytosis, or a marked anemia; accompanied anatomically by a hyperplasia of the lymphatic and glandular structures and clinically by a most wretched and agonising dyspnea.

This definition makes it plain that the one characteristic pathological feature of asthma is the unstableness of its blood. During long periods of quiescence the blood of the asthmatic usually presents no demonstrable pathognomonic constituents, but repeated blood analyses during attacks most vividly picture its unstableness. During some attacks the blood will present quite constant pathologic changes, indicating throughout the attack a decided lymphocytosis, an intestinal toxemic leukocytosis or an anemia, but not infrequently we find the blood rapidly oscillating between these three varieties, and occasionally there will be a blending of two of the three abnormalities or of the entire group.

A most noticeable feature of asthma when studied in this light is that it progresses in vicious circles. Thus we find the fundamental principle of asthma or an unstable blood, either inherited or acquired, circling from infancy to senility, and springing from this unstable blood at different ages there is a vicious circle differing from the preceding and each one marking an advance of the disease. So evident is this advent that an acute observer could draw a circle for every year's duration, but only a few of the most pronounced are here illustrated. At infancy this unstable blood manifests itself in a vicious circle beginning with a perverted metabolism, which results in a lymphocytosis, that in turn produces a clogging of the respiratory apparatus with lymph and mucus sufficient to cause the dyspnea.

Vicious Circles of Asthma.

During youth we observe an intestinal toxemia and perverted metabolism, lymphoanemia, adenoids and adenolymphoceles and dyspnea. At adult life it has progressed to intestinal toxemia and perverted metabolism, toxic leukocytosis and amyloid degeneration, enlarged laryngo-tracheal glands and dyspnea. In old age it terminates in a debilitated and nonbiomagnetic red blood corpuscle, anemia, impaired oxygenation and dyspnea.

The tendency of asthma, as it were, to progress in these vicious circles makes its pathology most interesting and fascinating, as it brings symmetry and definiteness out of chaos and confusion, which being as they are, in accord with nature, furnish another proof of its correctness.

### THE ASTHMATIC LYMPHOCYTOSIS.

In considering the pathology of this group we must bear in mind the immediate close and essential copartnership of digestion, oxygenation and metabolism, and at the same time recall the physiological facts that fat food products after their absorption by the lacteals travel in all a distance of only about 27 inches, through the thoracic duct, left innominate, superior vena cava, right heart and pulmonary arteries, direct to the lungs for oxygenation, separation and classification before passing through any important digestive organ for modification; also that the other food products as proteids, are taken up directly by the blood through the portal circulation, which after passing through the liver for modification, immediately reach the lungs for further important chemical metabolic preparation. This lung and thoracic food, lymph, serum and blood sifting or filtering process, together with oxygenation, is made possible and facilitated by the physiological peculiarity of the lymphatic system that some of its vessels end and begin by open mouths in the pleura, which, therefore, for present purposes may be regarded as vast local expansions of portions of the lymph path.

It then is evident that there is a filtration as well as an oxygenation of the blood in the lungs which permits it to enter the lungs as thick, blue, sluggish, venous blood, and to leave them as thin, scarlet, active, arterial blood. With these few, simple, common and well known but too infrequently discussed physiological facts as a working basis, the pathology of the lymphocytic asthma, as shown by the clinical and demonstrable features of the case, become a certainty. As would be expected the most serious forms of this variety of asthma develops during lactation, and most frequently in fat, flabby muscled and scurvy-like infants, or in infants that are reared among unhygienic surroundings.

Infants of this class that are taking on and rapidly absorbing and assimilating large amounts of milk, do not infrequently, from some slight digestive disturbance, have their metabolism thrown out of balance sufficiently to generate a lymphogenous chyle, that when carried to the blood acts more as a lymphagogue than a true hematogenic substance, and which when in turn is carried to the lungs for oxygenation and separation, nearly and in fatal cases actually drowns the infant in its own secretions. The clinical pictures of these cases carry with them a vast amount of valuable, open and unimpeachable evidence, that to the unbiased mind, furnish satisfactory proof of their lymphogenous origin, even without the clinching verdict of the microscope. The onset is sudden and with but very little warning. The child may give a few slight "shudders" twenty-four hours or so before an attack, and it is apt to be very peevish, worrisome, and possessed of an unquenchable thirst or desire to nurse, which the mother imprudently gratifies, thus hastening the on-coming attack.

The kidney, as the blood's ever diligent disposal plant and alert equilibrium preserve, is the first organ to detect the blood's dangerous condition and it endeavors to correct matters, hours before the attack, by filtering off as much as it possibly can of the useless material from the blood, which results in nearly a constant outpouring of a thin, pale odorless urine containing indican. During the attacks there is an extreme and alarming degree of cyanosis, and a dyspnea that nearly equals breathlessness. With each respiration mucus will bubble from the mouth in a frothy manner, and by turning the child over on its belly and shaking it, a thick, slimy mucus will string from its mouth. The digestive disturbance is manifested by flatulency and some vomiting. The kidney is working during the attack as it was preceding it, but after the attack the urine becomes scanty, high-colored and loaded with uric acid from the destruction of the white blood cells.

This alarming condition is almost instantly relieved by draining the lymph from the blood with epsom salts, and expelling the mucus from the respiratory apparatus. The blood count often shows as many as 94,300 white cells and usually 75 per cent. of these are lymphocytes. A vast majority of these lymphocytes are small, showing that they have come directly from the lymph channels and the chyle. Two of the above class of cases have, under my immediate observation, proved fatal, one of which I reported last October in a previous paper, suddenly expired in a severe cyanotic attack four months later. All other of my cases of this class I have thus far been able to rescue by a timely depletion.

In a few months after these severe cyanotic lymphogenous attacks, the child, not infrequently, develops the more constant lymphoanemic dyspnea, which, however, is always worse at night. The child soon becomes flabby-muscled, thin and pale, with a pronounced intestinal indigestion. From the first year onward this alarming variety is usually entirely substituted for the more chronic lymphoanemic form which frequently results in adenoids of the nasopharynx, adenolymphoceles, and more or less engorgement and hypertrophy of the lymph spaces of the entire respiratory tract. The two cardinal factors in the production of the asthmatic dyspnea when due to a lymphocytosis are: (1), the hemoglobin cannot reach the oxygen in the lungs; (2) the oxygen cannot reach the lungs.

The hemoglobin is handicapped in reaching the oxygen in the lungs, first, by its being diluted with and thickly surrounded by lymphocytes; and, second, by a thickened and air-tight condition of the partition between the oxygen and hemoglobin, due to an accumulation of mucus in the lung substance. The hemoglobin is also often in grave or fatal cases still further excluded from oxygen, by a collateral engorgement of the lung capillaries with lymph. The oxygen is debarred from reaching the lungs by their partially filling with mucus together with the trachea and larynx. In favorable cases, after an hour or so, the pathology changes and the lymph is gotten rid of by some being metamorphosed into normal blood substance, and the rest by elimination.

### ASTHMA DUE TO A TOXEMIC LEUKOCYTOSIS.

The pathology of this most important group is best studied in sections of its vicious circle, taken up in the order suggested by the clinical features and determined by laboratory investigations. As previously shown this group has an unstable blood to start with and one that perhaps has passed through the lymphocytic variety in infancy, and had its disintegrating spells, to be followed by its rapid but frail rebuilding periods till early adult life, when we find a blood that is extremely sensitive to toxins, and one that also has great difficulty in maintaining its metabolic equilibrium. With a case under constant observation the first thing of pathological interest after a prolonged period of freedom, and preceding an attack, will be a rapid alteration between constipation, a fetid diarrhea and flatulency. After a siege varying from ten days to three weeks of this mild intestinal indigestion and toxemia, enough toxins are absorbed by the blood, together with those generated in the blood itself from its own perverted metabolism, to start it on its catabolic career. The kidney is the first organ to take note of the blood changes which results in the characteristic polyuria and indicanuria. The leukocytes and eosinophiles soon show an appreciable increase. The clinical objective and subjective evidence of a disintegrating blood at this stage are an occasional swelling of hands, feet and eyelids; tired, achy, languid and chilly sensations; drowsiness after meals, irritableness; an unusual snugness of collar, hoarseness and difficulty in talking.

The intestinal toxemia progresses until the stools become small, frequent, acid fermative or alkaline cadaveric and possessed of a foul, irritating and penetrating odor that forces the inspector to turn away in disgust. This condition is soon followed by a pronounced chill lasting an hour or so, which, however, as a rule, has no febrile reaction. Following the chill the leukocytic count rapidly runs up to 50,000 or 60,000; 25 or 30 per cent. of which are, by a differential count, eosinophiles. After this count we can positively foretell the on-coming attack, for we now know we have a blood that has disintegrated and one that is loaded with a waste and useless material that must be gotten rid of, and we find that this is soon eliminated through the mucous membranes and expelled from the body in one or all of three ways,—namely, expectoration, diarrhea or vomiting.

The special, or if combined, principal route of elimination would be determined by both intrinsic and extrinsic etiological factors. The intrinsic factors would be (1), the excessively large number of dead leukocytes and other foreign matter in the blood, together with (2) the essential vital power that the blood has of separating itself from useless material, combined with (3) the fact that the only outlet or "dumping ground" for the blood is the mucous membranes. The extrinsic factors would be (1) the acquired and (2) the induced. Under the acquired we would have habitually an impaired vitality or chronic catarrhal condition of the mucous membrane of the region involved, which would constitute to the blood the line of least resistance, and therefore most naturally its chief "dumping ground." In the asthmatic we find this state of affairs in the mucous membrane of the respiratory tract.

About twenty-four hours after the chill the patient notices a pronounced bass-like change of voice and an unusual snugness of collar. The blood now has unloaded enough of its dead leukocytes and other useless material in the numerous glands situated in the larynx to cause a noticeable interference with respiration. In about two hours more the ventricular bands or false chords are so thickened and hypertrophied by an engorgement of the numerous wide lymph spaces found in them, which together with an engorgement of the many mucous glands located in the ventricles of the larynx and their ascending pouches, causes the ventricular bands to project into the lumen of the larynx far enough to catch sufficient air on expiration to produce a valvular action that nearly closes the air-tube. The little air that is forced out during the prolonged torturous and difficult expiration produces a loud whistling, wheezing sound coming directly from the larynx, as shown by both auscultation and palpation. This engorged condition of the larynx, although located high up, is extremely hard to view, owing to the fact that in depressing the tongue to observe it the ventricular bands are put on a tension which conceals and pushes back the engorged glands.

After about twenty-four hours of this laryngeal dyspnea, the glands undergo a resolution and degeneration, with the expulsion of thick mucus, which is expectorated in lumps the size of a pigeon's egg and of a tough, tenacious, viscid consistency, which gives it an appearance of fat tissue and enables one to pick it up and handle it about with the fingers. On inspection it is found to contain several little grayish pearly balls, which on being unraveled and viewed under the microscope are found to be composed of delicate, convoluted spirals (Curschmann's) made of numerous individual filaments. Other portions of the sputum under the microscope, without staining, are found to contain numerous leukocytes, exhibiting bright, yellowish, coarse granulations; among these are numerous colorless pointed octohedral crystals (Charcot-Leyden's). Disseminated throughout the field are numerous eosinophilic granules derived from ruptured eosinophile cells. This mucus responds very readily to amyloid tests thus denoting its starchy nature or amyloid degeneration. The blood while at all periods responding to the iodine test has a more pronounced iodophilia at this stage than any other.

While iodophilia generally indicates toxemia, it in the blood of the asthmatic seems to point more to an amyloid degeneration, for at our institute we clinically have long known that foods rich in starch agreed very poorly with the toxic asthmatic. This starchy degeneration of the blood or leukocytes is also indicated by the fact that all starch eating animals, as the horse, sheep, monkey, and the like, are subjected to asthma, while the strictly carniverous animals never have it. The origin of Curschmann's spirals, as I have shown in previous papers, is due to a plugging of the minute ducts of the small glands with dead leukocytes, until finally the tissue about the duct softens and gives way, which permits the expulsion of the filament-like plug. As these filament-like plugs individually exude from the numerous closely crowded minute ducts, into the lumen of the air-tube, their free ends are caught by the air as it passes out and in and twisted together, making the convoluted spirals. That this is the true origin of these spirals is still further proven by the fact that they are contained only in the mucus first expectorated, which would show that after the glands once become softened, and their minute ducts dilated, they expel their contents as fast as formed, without the

"duct plugging" or filament formation. The leucocytes, eosinophiles and octohedral crystals found in the sputum do, of course, come directly from the leucocytic blood.

After the softening of the laryngeal glands and the expectoration of their contents, the patient has a few hours of ease with normal respiration, when the dyspnea begins again, due to an engorgement of the glands of the trachea and bronchioles. The air now passes in and out through the larynx freely, and without any wheezing, to meet with obstructions farther down, as indicated by the location of the dry râles and the attitude of the patient. Owing to the blood's diminished alkalinity together with its leukocytic, toxic and amyloid condition, it is unable to utilise but a small portion of the oxygen that required such a strained muscular effort to furnish, hence the blood reaches the respiratory center in the medulla in a poorly oxygenated condition, which irritates the pneumogastric nerve, thus continually urging the diaphragm on to a still more rapid and strained muscular action, regardless of the quantity of oxygen the lungs may already contain, which tends to excessively dilate the lungs and produce a temporary emphysematous condition, that if long continued becomes more or less permanent.

The duration of this tubular obstructed dyspnea is about the same as that of the laryngeal, and it disappears by the same pathological process, throwing off the same thick, tenacious mucus which, however, seems to be expectorated in smaller sized lumps. With the beginning of the expectoration a constant cough develops, eliminating in a few hours over a pint of thick, sticky mucus, loaded with the white balls containing the asthma spirals. So thick is this mucus that when its receptacle is jarred the mass will tremble like so much jelly, and when diluted with water and emptied, it will adhere to the sides of its retainer and string down for nearly two feet, and if the vessel be turned back slowly a large amount of the mucus will draw back into it. Mucus of this nature is expectorated for about ten hours after which the spirals gradually disappear; with the disappearance of the spirals the expectorations become more profuse; the quantity of mucus expectorated at this period is appalling.

The cough is constant, and each cough usually results in the raising of as large a quantity of blood débris or mucus, rich with dead leukocytes and sweet tasting amyloid material, as could be forced into the mouth. While this pouring out of blood débris into the air-tubes, with its free expectoration, continues without interruption, there is but little if any dyspnea; but if for any reason the blood has its vitality reduced, as it is very apt to do when, for instance, deprived of the chemical and electrical effect of sun or daylight, as in the night, so that it does not readily separate itself from the dead leucocytes and other débris, the dyspnea will return, and the expectorations cease or become greatly diminished. A striking proof that the dyspnea at this time is largely due to an excessively large number of leukocytes in the blood is found in the fact that it will disappear like magic within twenty or thirty minutes after the administration of a large dose of quinine in solution. During the eliminating and expectorating stage, dyspneic spells of this nature will occasionally suddenly develop, coming on nearly as often during daylight as night darkness. When coming on in the daytime the dyspnea will usually appear an hour or so after the ingestion of food, or when the digestion leukocytosis is at its height. Not infrequently, however, quite severe attacks will come on suddenly, and without any warning, while the patient is up and about, due to an accumulation of mucus in the trachea, just as one's nose will become stuffed up with mucus during an acute coryza, sufficient to preclude the entrance of air. Such attacks usually last for only twenty minutes or an hour, the patient experiencing immediate relief with the first expectoration.

This elimination and coughing period lasts for about ten days when it gradually subsides. Here the process usually abates. When as in protracted cases, it is continued further, the enfeebled blood and scurvy-like condition next manifests itself by producing spongy gums with extravasations of blood and a rapid accumulation of sordes upon the teeth. In about twenty-four hours the process extends to the larynx and upon hawking a rusty colored mucus can be raised. From the larynx the process soon extends to the trachea, bronchi and bronchioles, resulting in a frequent cough that is accompanied by a feeling of soreness in the chest and the expectoration of a rusty colored sputum, that not infrequently contains streaks of clear blood. The expectoration of mucus of this alarming character lasts about five days and with its cessation the patients rapidly gain in health and vigor. In one case the blood here gave another beautiful illustration of its infirmity by the production of a purpura hemorrhagic-like condition of a patch of skin the size of one's whole hand, located in the right anterior lower portion of the chest. Toxins starting this unstable blood on its catabolic career do not alone originate in the intestinal canal or from the blood's own perverted metabolism, but they may be introduced from some outside source as the toxins of syphilis or some simple blood infection. A case now at the institute after being cured of his digestive metabolic toxemic asthma received a simple infection of the thumb which reëstablished the old vicious circle.

THE ASTHMATIC ANEMATOSIS.

At the origin of this circle we have an unstable blood plus an old, withered, deficient and nonbiomagnetic corpuscle, that has withstood the vicissitudes of the other varieties of asthma, or has passed through a prolonged siege of malarial fever, lead poisoning, tertiary syphilis, or other blood debilitating process. This nonbiomagnetic and debilitated blood is at all times playing close to the oxygenating margin, yet at favorable periods, such as during a sun or electric light bath, owing probably largely to their magnetic influence on the blood, or following a cold sponge bath, or altitude owing to an increase in numbers, or a crowding together of the red blood corpuscles, there will be periods of no asthma. These short, sometimes only hourly periods of freedom, are suddenly followed by an attack, from some slight blood debilitating influence, as the lack of sunlight in the night, increased atmospheric humidity, south winds, ground dampness, or swampy districts, sudden climatic changes, over exertion, emotions, digestion leukocytosis, and numerous other causes to which this delicate and sensitive blood pathologically reacts.

Thus, owing to some individual peculiarity, we may find the attacks coming on night after night in one, after meals in another, during atmospheric changes in still another and so on, yet each and every cause of which, as we can now demonstrate produces a blood change sufficient to throw this delicately balanced blood over its oxygenating margin. The blood's inability to properly perform its oxygenating function, in the asthmatic anematosis, is not alone due to a loss of hemoglobin as in other anemias, but it is due more to the hemoglobin's unfavorable environments, or the asthmatic's abnormal blood biochemy. These unfavorable hemoglobin environments are:

First. A diminution in the blood's alkalinity, said normal alkalinity constituting the basic principle of its oxygenation.

Second. A diminution in and impaired vitality of corpuscle substance as found in all anemias.

Third. A slight impairment of the rouleux formation, due to the toxic plasma.

Fourth. A diminution in animal electromagnetism.

There are many simple yet positive proofs that the dyspnea is in part, due to a deficient biomagnetism.

There is no loud wheezing, tubular obstruction, or Curchsmann's spirals in the purely anemic dyspnea, and their appearance would indicate a complication with the toxic leukocytic variety, which not infrequently occurs. As in the preceding varieties the kidney is first to detect the blood changes, giving the characteristic variable asthmatic urine. It is hoped that enough facts as collected and proven from time to time have here been given to wrest this truly torturous malady from the realms of mystery and quackism, and to safely land it in the hands of conservative physicians.

261 Georgia Street.

## Figures and Tables

**Figure f1:**